# Lack of Evidence of Severe Acute Respiratory Syndrome Coronavirus 2 (SARS-CoV-2) Spillover in Free-Living Neotropical Non-Human Primates, Brazil

**DOI:** 10.3390/v13101933

**Published:** 2021-09-25

**Authors:** Lívia Sacchetto, Bárbara Aparecida Chaves, Edson Rodrigues Costa, Aline Souza de Menezes Medeiros, Marcelo Gordo, Danielle Bastos Araújo, Danielle Bruna Leal Oliveira, Ana Paula Betaressi da Silva, Andréia Francesli Negri, Edison Luiz Durigon, Kathryn A. Hanley, Nikos Vasilakis, Marcus Vinícius Guimarães de Lacerda, Maurício Lacerda Nogueira

**Affiliations:** 1 Laboratório de Pesquisas em Virologia, Departamento de Doenças Dermatológicas, Infecciosas e Parasitárias, Faculdade de Medicina de São José do Rio Preto, São José do Rio Preto 15090-000, Brazil; liviasacchetto@gmail.com; 2 Instituto de Pesquisas Clínicas Carlos Borborema, Fundação de Medicina Tropical Doutor Heitor Vieria Dourado, Manaus 69040-000, Brazil; bachaves89@gmail.com (B.A.C.); edsonzoo@hotmail.com (E.R.C.); alinesouza_mm@hotmail.com (A.S.d.M.M.); 3 Programa de Pós-Graduação em Medicina Tropical, Universidade do Estado do Amazonas, Manaus 69040-000, Brazil; 4 Programa de Pós-Graduação em Ciências da Saúde, Universidade Federal do Amazonas, Manaus 69020-160, Brazil; 5 Laboratório de Biologia da Conservação, Projeto Sauim-de-Coleira, Instituto de Ciências Biológicas, Universidade Federal do Amazonas, PPGZOO, PPGCASA, CAPES (Coordenação de Aperfeiçoamento de Pessoal de Nível Superior), Manaus 69080-900, Brazil; projetosauim@gmail.com; 6 Departamento de Microbiologia, Instituto de Ciências Biomédicas, Universidade de São Paulo, São Paulo 05508-000, Brazil; daniellebastos@yahoo.com.br (D.B.A.); danibruna@gmail.com (D.B.L.O.); eldurigo@usp.br (E.L.D.); 7 Hospital Israelita Albert Einstein, São Paulo 05652-900, Brazil; 8 Centro de Inovação e Desenvolvimento, Instituto Butantã, São Paulo 05503-900, Brazil; 9 Departamento de Vigilância Epidemiológica de São José do Rio Preto, São José do Rio Preto 15084-010, Brazil; anabetaressi@gmail.com (A.P.B.d.S.); andreiafrancesli@hotmail.com (A.F.N.); 10 Plataforma Científica Pasteur, Universidade de São Paulo, São Paulo 05508-020, Brazil; 11 Department of Biology, New Mexico State University, Las Cruces, NM 88003, USA; khanley@nmsu.edu; 12 Department of Pathology, The University of Texas Medical Branch, Galveston, TX 77555, USA; nivasila@utmb.edu; 13 Sealy Center for Vector-Borne and Zoonotic Diseases, University of Texas Medical Branch, Galveston, TX 77555, USA; 14 Center for Biodefense and Emerging Infectious Diseases, University of Texas Medical Branch, Galveston, TX 77555, USA; 15 Center for Tropical Diseases, University of Texas Medical Branch, Galveston, TX 77555, USA; 16 Institute for Human Infection and Immunity, University of Texas Medical Branch, Galveston, TX 77555, USA; 17 Instituto Leônidas e Maria Deane, Fiocruz, Manaus 69057-070, Brazil

**Keywords:** coronavirus, emerging virus, spillback, non-human primates, COVID-19

## Abstract

Severe acute respiratory syndrome coronavirus 2 (SARS-CoV-2), the agent of coronavirus disease 2019 (COVID-19)**,** is responsible for the worst pandemic of the 21st century. Like all human coronaviruses, SARS-CoV-2 originated in a wildlife reservoir, most likely from bats. As SARS-CoV-2 has spread across the globe in humans, it has spilled over to infect a variety of non-human animal species in domestic, farm, and zoo settings. Additionally, a broad range of species, including one neotropical monkey, have proven to be susceptible to experimental infection with SARS-CoV-2. Together, these findings raise the specter of establishment of novel enzootic cycles of SARS-CoV-2. To assess the potential exposure of free-living non-human primates to SARS-CoV-2, we sampled 60 neotropical monkeys living in proximity to Manaus and São José do Rio Preto, two hotspots for COVID-19 in Brazil. Our molecular and serological tests detected no evidence of SAR-CoV-2 infection among these populations. While this result is reassuring, sustained surveillance efforts of wildlife living in close association with human populations is warranted, given the stochastic nature of spillover events and the enormous implications of SARS-CoV-2 spillover for human health.

## 1. Introduction

Severe acute respiratory syndrome coronavirus 2 (SARS-CoV-2) was first identified in December 2019 after reports of acute respiratory syndrome outbreak in patients in Wuhan, Hubei Province, China, and rapidly spread worldwide, ushering in the COVID-19 pandemic [1,2]. As of 23 July 2021, a total of 192,284,207 laboratory-confirmed SARS-CoV-2 cases and 4,136,518 deaths have been reported by the WHO in 242 countries [3]. Along with its impact on public health, the pandemic has radically disrupted human activities and resulted in severe socioeconomic damage worldwide [4].

Coronaviruses (CoVs) (order: *Nidovirales*, family: *Coronaviridae*, subfamily: *Coronavirinae*) are known to infect a wide variety of birds (*Gammacoronaviruses* and *Deltacoronaviruses*) and mammals (predominantly *Alphacoronaviruses* and *Betacoronaviruses*). Moreover, this family of viruses exhibits a propensity for host switching: the seven known human coronaviruses (HCoV-OC53, HCoV-229E, HCoV-NL63, HCoV-HKU1, SARS-CoV-1, MERS-CoV, and SARS-CoV-2) all originated via spillover of ancestral viruses maintained in non-human animal hosts [5]. Of these, the three highly pathogenic human CoVs (SARS-CoV-1, MERS-CoV, and SARS-CoV-2) likely emerged from bats, albeit this process was facilitated by intermediate hosts [5,6,7].

Given the evolutionary lability of CoVs, there has been considerable concern about spillover of SARS-CoV-2 from humans into additional species since the inception of the pandemic [8], and indeed surveillance has revealed SARS-CoV-2 infection of individuals of a wide variety of animal species in domestic, farm, and zoological park environments [9,10,11]. Alarmingly, significant virus transmission within some of these species has been documented, particularly farmed mink [12]. SARS-CoV-2 has also spilled over from infected, farmed mink into feral mink populations [13] as well as domestic cats and dogs [14]. Very recently, dogs and cats infected by human contacts with SARS-CoV-2 variants of concern (VOC) have been detected [15]. 

In addition to these natural infections, experimental studies have shown that many animal species, including cats, ferrets, raccoon dogs, deer, rabbits, fruit bats, and hamsters, are susceptible to SARS-CoV-2 infection [16,17,18]. Of particular relevance for this study, several non-human primates (NHPs) have been found to be susceptible to experimental infection with SARS-CoV-2 and to recapitulate at least some of the symptoms of COVID-19 in humans [19,20], including one neotropical monkey, the common marmoset *Callithrix jacchus* [19,21]. *C. jacchus* has also proven a useful model for MERS-CoV [18,22] as well as SARS-CoV [23]. Machine learning methods also highlight primates generally, and *C. jacchus* specifically, as having a high potential to sustain SARS-CoV-2 transmission [24]. 

Together, these findings on natural and experimental infection ring a warning bell about potential spillover of SARS-CoV-2 to free-living neotropical monkeys living in close proximity to humans. If such spillover led to sustained transmission within these monkeys, this new transmission cycle would greatly complicate efforts to control SARS-CoV-2 in humans and could represent a catastrophe for conservation of neotropical non-human primates [8,25]. Thus, the current study investigated evidence for SARS-CoV-2 spillover into NHPs in two regions of Brazil, namely, Manaus and São José do Rio Preto, that have experienced prolonged, high-intensity transmission of SARS-CoV-2.

## 2. Materials and Methods

### 2.1. Study Areas

**Figure 1:**
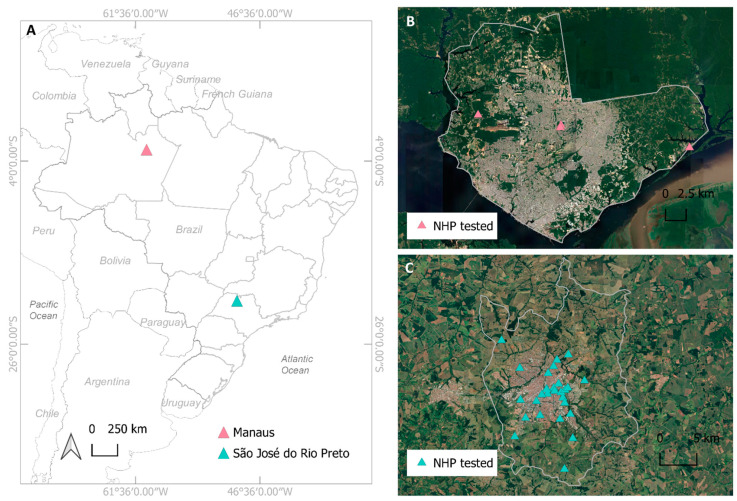
Location of non-human primate (NHP) sampling. (**A**) Geopolitical map of Brazil, highlighting the two sample municipalities, Manaus (pink triangle) and São José do Rio Preto (green triangle). (**B**) Satellite image of Manaus site showing the distribution of the NHP tested (pink triangles). (**C**) Satellite image of São José do Rio Preto site showing the distribution of the NHP tested (green triangles). Maps were created using the QGIS software version 3.8.2.

Manaus, a city in the state of Amazonas and São José do Rio Preto (SJdRP), a city in the state of São Paulo, are two major foci of SARS-CoV-2 transmission in Brazil (Figure 1A). NHP surveillance programs had already been established in both cities prior to the onset of the SARS-CoV-2 pandemic through the Coordinating Research on Emerging Arboviral Threats Encompassing the Neotropics (CREATE-NEO) project, a member of The Centers for Research in Emerging Infectious Disease (CREID) network. Manaus is the capital of the state of Amazonas (427 km^2^ of urban area) with 1,802,014 inhabitants, located in the heart of the Amazon basin, the world’s largest tropical rainforest (Figure 1B). São José do Rio Preto (SJdRP) is located at the northwestern region of the state of São Paulo (9681 km^2^ of urban area), with 408,258 inhabitants. Two biomes cover the area: Cerrado and the Atlantic Forest (Figure 1C). Importantly, both cities encompass multiple forest parks in which NHPs live at high densities and which are visited by large numbers of people daily.

### 2.2. Ethics

This study was approved by the System of Authorization and Information on Biodiversity (SISBIO), protocol number: 57003-7, issued 27 July 2017. The protocol was submitted and approved by the Committee on Animal Ethics (CEUA) of the Fundação de Medicina Tropical Doutor Heitor Vieira Dourado (FMT-HVD) (protocol number: 003188, issued 30 October 2017), as well as by the Institutional Animal Care and Use Committee of the University of Texas Medical Branch (UTMB) (protocol no. 1706039A, re-issued: 1 June 2020).

### 2.3. Non-Human Primate Sampling

In SJdRP, a total of 34 carcasses of free-living NHPs in the genera *Callithrix, Callicebus*, and *Alouatta* (identification to the species level was not captured at the time of collection) (Table 1) found dead were collected from December 2020 to April 2021 (Figure 1C). Collection of NHP carcasses was performed under the auspices of the national yellow fever surveillance program. NHPs were necropsied by the city of SJdRP public health department, and biological samples, including brain, lung, liver, heart, kidney, and clot, were collected, and forwarded to the *Laboratório de Pesquisas em Virologia* (LPV). Epidemiological data were recorded for each specimen in the National Injury Information Notification System (SINAN). The samples were kept at −70 °C until further investigation.

In Manaus, 26 free-living *Saguinus bicolor* (pied tamarin) were sampled from July to November 2020 (Table 2) in three areas (Puraquequara, Tarumã and parque Municipal do Sumaúma) (Figure 1B) where NHPs are commonly found in close proximity to humans. Tomahawk Live Traps (Forestry Suppliers, Inc, Jackson, MS, USA) baited with bananas were used to capture the monkeys. Procedures and animal manipulations were performed to minimize any potential discomfort, distress, or pain by trained veterinary and research staff. Prior to any procedure, animals were anesthetized by intramuscular injection with ketamine hydrochloride (2.0 mg/kg) and were tagged with subcutaneous microchips (PRO ID™ Standard Chip, Covington, LA, USA) for future tracking and identification. All animals were housed individually in cages and held overnight and released early morning the next day at the capture sites. Blood samples were collected by femoral vein puncture, and samples were then transported to the laboratory under refrigeration (2–8 °C) and centrifuged for 10 min at 2000 rpm, and sera were stored at −80 °C until processing. 

### 2.4. Virus RNA Extraction and Real Time RT-PCR

Total RNA extracted from the liver samples of NHPs from SJdRP, which were collected as carcasses, was screened using the one-step real-time polymerase chain reaction (RT-qPCR) for YFV, ZIKV, DENV, and CHIKV. The Trioplex quantitative polymerase chain reaction (qPCR) assay was performed using a kit provided by the Centers for Disease Control and Prevention using primers and probes specifically designed for the detection of ZIKV, chikungunya virus (CHIKV), and all DENV serotypes [26], whereas for YFV, the assay described in Domingo et al. [27] was utilized. Briefly, 10 µL of vRNA, 0.5 µM of each probe and primer mixed, 12.5 µL the 2X PCR Master Mix, and 0.5 µL Superscript III RT/Platinum Taq enzyme mix (SuperScript^®^ III Platinum^®^ One-Step qRT-PCR System, Invitrogen, Carlsbad, CA, USA) were applied to a 96-well plate using the QuantStudio™ Dx instrument (Thermo Fisher Scientific, Waltham, MA, USA) with the following conditions: 50 °C for 30 s, followed by 45 cycles of 95 °C for 15 s, and 60 °C for 1 min. Results were interpreted as positive with cycle threshold (Ct) values less than 38.

After negative results for these arboviruses were observed, we decided to investigate the lung samples for the SARS-CoV-2 genome due to the proximity of the NHP population to humans and the ongoing COVID-19 pandemic. Total RNA was extracted from approximately 30 mg of lung using RNeasy Minikit (Qiagen, Germantown, MD, USA) according to the manufacturer’s instructions. All tissue handlings were performed in a laminar flow cabinet. RNA samples were screened for SARS-CoV-2 RNA using one-step real-time polymerase chain reaction (RT-qPCR) using primers and probes targeting the envelope (E), and nucleocapsid (N) regions of SARS-CoV-2 genome (GeneFinder COVID-19 Plus RealAmp Kit (OSANG Healthcare, KOR); sequences of primers and probes are proprietary and not provided by the manufacturer; https://www.fda.gov/media/137116/download accessed on 21 July 2021)). The RT-qPCR amplification was performed in a QuantStudio 3 Real-Time PCR System (Thermo Fisher Scientific, Waltham, MA, USA), and the conditions were as follows: 50 °C for 20 min for the reverse transcription, 95 °C for five minutes for pre-denaturation, followed by 45 cycles of denaturation at 95 °C for 15 s, and annealing at 58 °C for 60 s. The results were visualized in QuantStudio 3 software v1.5.1. The results were interpreted as being positive when the cycle threshold (Ct) values were less than 40; Ct > 40 or undetermined were interpreted as negative. The positive controls used in the assay are included in the GeneFinder kit and are comprised of two individual non-infectious DNA plasmids coding for the E gene and the N gene. The Ct values for the positive controls were 17.7 for the E gene and 16.8 for the N gene.

### 2.5. Plaque Reduction Neutralization Test (PRNT)

The Vero cell line, clone CCL-81 (ATCC, Manassas, VA, USA), was maintained in Eagle’s minimum essential medium (GIBCO, ThermoFisher Scientific, Waltham, MA, USA) at 37 °C in a humidified chamber containing 5% CO_2_. Growth media were supplemented with 10% FBS (GIBCO, ThermoFisher Scientific, Waltham, MA, USA), 100 U/mL penicillin, and 100 μg/mL streptomycin (GIBCO, ThermoFisher Scientific, Waltham, MA, USA). Serum samples were inactivated at 56 °C for 60 min and assayed to determine the specific neutralization antibody titers to SARS-CoV-2, on the basis of a modified protocol described previously [28]. Briefly, PRNTs were performed in 24-well microplates with 80–90% confluent Vero cells per well, using a fixed SARS-CoV-2/SP02.2020.HIAE.Br [29] virus inoculum (800 pfu/mL, *corresponding to 60–80 plaques/well for both negative control and naive serum*) against varying serum dilutions (1:20 to 1:2560). After one hour adsorption at 37 °C, plates were overlaid with a semi-solid medium (MEM 1X, 1% FBS, 1.5% carboxymethylcellulose) and incubated at 37 °C in 5% CO_2_ for 3 days. After this, the cell monolayer was fixed with 10% formalin solution and stained with amido black (Naphthol Blue Black, Sigma-Aldrich, St. Louis, MO, USA). PRNT titers were scored as the reciprocal of the highest dilution of serum that inhibited 90% of plaques (PRNT90). Samples scored as PRNT90 < 20 were considered negative. PRNT assays were performed in a biosafety level 3 of containment (BSL3), following WHO recommendations. 

## 3. Results

We screened the lung samples from 34 free-living NHP carcasses collected from December 2020 to April 2021 in SJdRP. All animals tested were negative for the presence of SARS-CoV-2 RNA (Table 1). All the serum samples collected from 26 free-living pied tamarin captured in Manaus between July and November 2020 and tested by the PRNT assay were negative for exposure to SARS-CoV-2 (Table 2).

## 4. Discussion

Since late February 2020, when the first official confirmed case of COVID-19 was reported in Brazil, the new coronavirus spread rapidly throughout the country. As of 23 July 2021, Brazil is one of the countries most affected by the ongoing pandemic with 19,473,954 confirmed cases and 545,604 deaths [30]. Given the close association between humans and NHPs in urban areas of Brazil, we decided to leverage our existing NHP surveillance network to survey for SARS-CoV-2 spillover into local monkeys. We captured or received samples from 60 free-living Neotropical NHPs belonging to four genera, *Saguinus*, *Callithrix*, *Alouatta*, and *Callicebus*, in two hotspots of COVID-19 in the states of São Paulo and Amazonas. Monkeys in the genus *Saguinus* were captured live, subjected to blood sampling, and re-released. Thus, these animals were screened for previous exposure to SARS-CoV-2 via a serological assay. Monkeys from the remaining genera (*Callithrix*, *Alouatta*, and *Callicebus*) were received as carcasses, and their lungs were assayed for active SARS-CoV-2 using molecular methods. No evidence of SARS-CoV-2 infection was detected in either group. One limitation of the study is the limited number of animals sampled; however, given our sample size of 34 animals in SJdRP, our power to detect SARS-CoV-2 at 5% prevalence was approximately 80% within the monkey population of the region, assuming that the size of that population does not greatly exceed 100,000 animals.

Although these results are reassuring, the threat of SARS-CoV-2 spillover persists, particularly in Brazil, which sustains a very high level of SARS-CoV-2 transmission [31]. Currently, SJdRP is classified as the third municipality in the number of COVID-19 confirmed cases in São Paulo state, totaling 89,130 cases, 2636 deaths, and a lethality rate of 3.0% [32]. Manaus, the capital and largest municipality of Amazonas, ranks first in the state in COVID-19 confirmed cases. According to the last epidemiological bulletin of the Amazonas state, Manaus recorded 194,957 COVID-19 cases, 9263 deaths, and a lethality rate of 4.8% [33]. Moreover, several SARS-CoV-2 VOCs circulate broadly in Brazil [34,35]. VOCs are SARS-CoV-2 lineages associated with increased transmissibility and severity and decreased vaccination effectiveness in humans. Whether these phenotypes also enhance potential for spillover into other animals is not yet known; nonetheless, their rapid dissemination in Brazil raises merits additional scrutiny of potential spillover hosts. 

Recent infections of zoo animals with SARS-CoV-2 that are not directly handled by keepers, such as lions, tigers, and gorillas [25,36,37], as well as evidence suggesting that SARS-CoV-2 may be circulating in free-living deer in the United States [38], underscore the potential for this virus to be transmitted at distances achieved during the closest approach between humans and NHPs in urban parks. Thus, continued surveillance of NHPs in this region is warranted and may identify geographic areas or particular NHP species prone to spillover, thereby helping to prevent further epidemics. Such surveillance falls within the mandate of the CREATE-NEO and confers the added benefit of generating samples for more general surveillance of pathogens with potential for emergence from NHPs into humans [39]. 

## Figures and Tables

**Table 1:** Characteristics of neotropical non-human primates investigated in São José do Rio Preto.

ID	Genus	Neighborhood	Date	SARS-CoV-2 RT-qPCR
NHP21/01	*Callithrix*	Engenheiro Schimdt	03/16/2020	Neg
NHP21/02	*Callithrix*	Vila Elvira	03/26/2020	Neg
NHP21/03	*Callithrix*	Central	04/17/2020	Neg
NHP21/04	*Callithrix*	Central	06/01/2020	Neg
NHP21/05	*Callithrix*	Jaguaré	06/08/2020	Neg
NHP21/06	*Callithrix*	Cidade Jardim	06/16/2020	Neg
NHP21/07	*Callithrix*	Vila Toninho	08/25/2020	Neg
NHP21/08	*Callithrix*	Americano	08/27/2020	Neg
NHP21/09	*Callithrix*	Vila Toninho	09/08/2020	Neg
NHP21/10	*Callithrix*	Caic	09/24/2020	Neg
NHP21/11	*Callithrix*	Vila Toninho	10/02/2020	Neg
NHP21/12	*Callithrix*	Solo Sagrado	10/06/2020	Neg
NHP21/13	*Callithrix*	Vila Toninho	10/21/2020	Neg
NHP21/14	*Callithrix*	Jaguaré	10/21/2020	Neg
NHP21/15	*Callithrix*	Jaguaré	10/23/2020	Neg
NHP21/16	*Callithrix*	Jaguaré	10/26/2020	Neg
NHP21/17	*Callithrix*	Solo Sagrado	10/27/2020	Neg
NHP21/18	*Callithrix*	São Francisco	11/06/2020	Neg
NHP21/19	*Callithrix*	Anchieta	11/09/2020	Neg
NHP21/20	*Alouatta*	Solo Sagrado	11/09/2020	Neg
NHP21/21	*Callicebus*	São Deocleciano	11/11/2020	Neg
NHP21/22	*Callithrix*	Americano	11/13/2020	Neg
NHP21/23	*Callithrix*	Americano	11/13/2020	Neg
NHP21/24	*Callithrix*	São Francisco	11/17/2020	Neg
NHP21/25	*Callithrix*	Central	12/10/2020	Neg
NHP21/26	*Callithrix*	Novo Horizonte	09/22/2020	Neg
NHP21/27	*Alouatta*	Jose Bonifácio	10/05/2020	Neg
NHP21/28	*Alouatta*	Tabapuã	10/26/2020	Neg
NHP21/29	*Callithrix*	Parque Industrial	02/03/2021	Neg
NHP21/30	*Callithrix*	Americano	02/11/2021	Neg
NHP21/31	*Callithrix*	São Francisco	02/17/2021	Neg
NHP21/32	*Callithrix*	Central	03/11/2021	Neg
NHP21/33	*Callithrix*	Central	03/18/2021	Neg
NHP21/34	*Callithrix*	Central	03/18/2021	Neg

**Table 2:** Characteristics of neotropical non-human primates investigated in Manaus.

ID	Genera/Specie	Neighborhood	Date	PRNT Titer
H111P-SB	*Saguinus bicolor*	Sumauma	10/11/2020	<20
H114P-SB	*Saguinus bicolor*	Sumauma	10/11/2020	<20
H115P-SB	*Saguinus bicolor*	Sumauma	10/11/2020	<20
H116P-SB	*Saguinus bicolor*	Sumauma	10/11/2020	<20
H117P-SB	*Saguinus bicolor*	Sumauma	10/11/2020	<20
H128P-SB	*Saguinus bicolor*	Puraquequara	21/07/2020	<20
H129P-SB	*Saguinus bicolor*	Puraquequara	21/07/2020	<20
H130P-SB	*Saguinus bicolor*	Puraquequara	21/07/2020	<20
H131P-SB	*Saguinus bicolor*	Puraquequara	21/07/2020	<20
H132P-SB	*Saguinus bicolor*	Taruma	11/08/2020	<20
H133P-SB	*Saguinus bicolor*	Taruma	11/08/2020	<20
H134P-SB	*Saguinus bicolor*	Taruma	11/08/2020	<20
H135P-SB	*Saguinus bicolor*	Taruma	11/08/2020	<20
H136P-SB	*Saguinus bicolor*	Taruma	11/08/2020	<20
H137P-SB	*Saguinus bicolor*	Taruma	11/08/2020	<20
H138P-SB	*Saguinus bicolor*	Sumauma	09/11/2020	<20
H139P-SB	*Saguinus bicolor*	Sumauma	09/11/2020	<20
H140P-SB	*Saguinus bicolor*	Sumauma	09/11/2020	<20
H141P-SB	*Saguinus bicolor*	Sumauma	09/11/2020	<20
H142P-SB	*Saguinus bicolor*	Sumauma	09/11/2020	<20
H143P-SB	*Saguinus bicolor*	Sumauma	09/11/2020	<20
H144P-SB	*Saguinus bicolor*	Sumauma	09/11/2020	<20
H145P-SB	*Saguinus bicolor*	Sumauma	09/11/2020	<20
H146P-SB	*Saguinus bicolor*	Sumauma	09/11/2020	<20
H147P-SB	*Saguinus bicolor*	Sumauma	09/11/2020	<20
H148P-SB	*Saguinus bicolor*	Sumauma	09/11/2020	<20

PRNT: plaque reduction neutralization test.

## Data Availability

Original data are available from the authors upon request.
